# Relationships among sport participation, sport and social competence, and mental health symptomatology

**DOI:** 10.3389/fspor.2026.1833949

**Published:** 2026-05-26

**Authors:** Dawn Anderson-Butcher, Samantha Bates, Anthony J. Amorose, Simon Quick

**Affiliations:** 1 College of Social Work, The Ohio State University, Columbus, OH, United States; 2 School of Kinesiology and Recreation, Illinois State University, Normal, IL, United States; 3 School of Sport, Rehabilitation and Exercise Sciences, University of Essex, Colchester, United Kingdom

**Keywords:** adolecence, mental health symptomatology, social competence, sport competence, sport participation

## Abstract

**Introduction:**

Rising rates of adolescent mental health symptomatology have prompted increased attention to the potential protective role of sport participation. While sport participation has the potential to support positive mental health outcomes, such as lower levels of anxiety and depression, athletes may also experience unique stressors and pressures surrounding sport. Such dualities underscore the need to better understand the mechanisms underlying the relationship between sport participation and mental health outcomes.

**Methods:**

This study used structural equation modeling to examine whether sport participation (61% of the sample) was related to reduced internalizing and externalizing behaviors among a sample of 628 adolescents aged 13–18 (*M* = 14.55; *SD* = 1.44) and explored whether these relationships were mediated by perceived sport and social competence.

**Results:**

The hypothesized model demonstrated good fit (RMSEA = .043, SRMR = .044). Sport participation was positively associated with sport competence (β = .74) and social competence (β = .24). Internalizing behaviors were negatively predicted by sport (β = −.27) and social competence (β = −.14), and externalizing behaviors were negatively predicted by social competence (β = −.33) and positively predicted by sport competence (β = .11). A significant indirect pathway emerged from sport participation to internalizing behaviors (total indirect β = −.23) primarily through sport competence (β = −.20), whereas social competence did not uniquely mediate this relationship. Indirect effects for externalizing behaviors were non-significant. Measurement invariance analyses indicated that the model was equivalent in relationships across female and male adolescents.

**Discussion:**

Findings highlight sport competence as a key mechanism through which sport participation may relate to lower internalizing symptomatology and suggest that enhancing competence experiences in sport may be an important target for mental health promotion.

## Introduction

Adolescent mental health concerns in the United States have increased substantially, with heightened risk during developmental periods characterized by shifts in peer and family relationships, identity formation, and social-emotional skill development (1–4). Organized sport is a key social context that can mitigate developmental risks and foster protective factors, equipping young people with skills to navigate both sport and life. Consistent with this view, organized sport, defined as a physical activity involving exertion and skill coupled with competition against others, has been associated with positive mental health outcomes and broader indicators of well-being among adolescents (5–8).

Adolescents who participate in sport and engage in higher levels of physical activity report fewer symptoms of hyperactivity, depression, and anxiety, as well as fewer peer and conduct problems (5, 9, 10). Physical fitness, often a byproduct of sport participation, also has been shown to reduce symptoms of loneliness and depression and lead to greater cognitive, social, and athletic competence, and overall self-worth (11). Further, a recent meta-analysis found small effect sizes for a binary measure of sport participation on both depression and anxiety such that youth who participated in sport reported fewer mental health symptoms (12). Additionally, a small negative effect was found between sport participation and depression, further providing support for its role in supporting mental health and well-being. Collectively, these findings suggest sport participation can serve as a meaningful context for supporting adolescent mental health.

However, these benefits are not uniform, and growing evidence suggests that sport participation may also introduce unique stressors into adolescents' lives. Indeed, the mental health implications of sport are highly dependent upon how sport is structured, the climate created by coaches and organizations, and the demands experienced by athletes. Unfortunately, during adolescence, a period already characterized by increased vulnerability, sport participation may foster heightened risks as young people are juggling a desire for more independence while also balancing academic responsibilities and athletic commitments (13–16). In fact, a substantial proportion of high school student-athletes report difficulty managing school and sport demands (13). Social influences including parents and coaches, pressures to perform and specialize, and win-at-all-costs cultural norms also serve as additional stressors (14). Accordingly, sport participation may not be inherently protective.

Rather, the current body of evidence suggests sport has the capacity to create conditions for both resilience and risk. Although moderate levels of stress and arousal may enhance performance, chronic stress and prolonged exposure to adversity can contribute to mental and emotional health difficulties (17, 18). When combined with sport-specific demands such as intensive training loads, fatigue, and time constraints, adolescents may face elevated risk for depression, anxiety, substance misuse, aggressive behavior, and burnout (14). At the same time, other research suggests sport participation may buffer against certain difficulties, including social anxiety (19). The divergent findings underscore the importance of identifying the mechanisms linking sport participation with internalizing and externalizing symptomatology (20). The present study responds to this call by examining perceived sport competence and social competence as potential explanatory pathways through which sport participation relates to adolescent mental health outcomes.

## Sport participation, competence, and mental health symptomatology

A key aspect of sport as a developmental context is its emphasis on competence, defined as the skills, abilities, and knowledge that athletes perceive themselves as possessing across domains critical to performance (21). Grounded in Harter's (22) competence motivation model, two domains are particularly salient in sport settings: sport and social competence (23–25). Sport competence reflects perceptions of mastery of sport-specific tactics and techniques over time. Social competence encompasses intra- (e.g., emotional regulation) and inter-personal skills (e.g., communication) that are essential for the successful mastery of skills, managing performance, and during competition (26).

A growing body of research in traditional youth sport (27), physical activity settings (28), and sport-based youth development programming (25, 29) demonstrates how perceptions of sport competence can improve with sport involvement. In turn, sport competence also may have implications for broader mental health, particularly in relation to reducing internalizing and externalizing symptoms (e.g., anxiety, depression, etc.). Carter and colleagues (30) used path analyses to explore the relationships among sport participation, frequency of participation, sport competence, and internalizing symptomatology among a diverse adolescent sample. Researchers found that although greater frequency of participation predicted higher levels of anxiety and depressive symptoms (although categorical sport participation was not related to symptoms), sport competence was the strongest predictor of mental health, with higher levels perceived competence linked to lower internalizing symptomatology. Findings suggest that participation in sport alone may not support or undermine mental health; rather, adolescents' perceptions of competence within sport may represent a critical explanatory mechanism.

Evidence also is mounting regarding sport participation's role in promoting social competence. A recent randomized design study demonstrated significant growth in parent-reported social competence among youth participating in a sport-based positive youth development program compared with non-participating peers (31). Research in traditional sport settings further attests to these relationships. Bedard et al. (32) followed youth over four years and found higher levels of sport participation were associated with small gains in social competence in late childhood and early adolescence, suggesting athletes may internalize positive self-perceptions through relationships with coaches, teammates, and others involved in sport. Similarly, Caldarella et al. (33) reported that adolescents who participated in sport exhibited higher levels of parent-reported resilience, inclusive of social competence, self-regulation, responsibility, and empathy, than those not involved. This body of work links sport participation to the development of social competence, a construct consistently associated with improved adaptive functioning and lower internalizing symptoms (34, 35).

Given these established links, an important next step is determining whether sport and social competence function as mechanisms through which sport participation relates to adolescent mental health. Empirical tests of mediation remain limited, yet some preliminary findings support this possibility. For instance, Qian and colleagues (36) identified social-emotional skills as mediators in the relationship between sport participation and mental well-being (as measured by cheerfulness, calmness, and having a positive outlook). Researchers further noted a direct effect between sport participation and positive mental well-being. Additionally, Hoffmann and colleagues (37) reported that team sport participation was associated with lower internalizing, social, cognitive, and attention-related difficulties compared with non-participation. Notably, variations emerged by sport type and gender. Team sport participation was linked to reduced rule-breaking behaviors among females, whereas participation in individual sports was associated with higher levels of several mental health difficulties. As seen here, the mental health implications of sport differed depending on the context and structure of participation.

Despite these advances, few studies have explicitly examined mediating processes, particularly models that include both sport competence and social competence within the same analytic framework (24). Moreover, much of the existing literature is constrained by modest sample sizes, cross-sectional designs, or measurement limitations (32). As a result, the mechanisms linking sport participation to both internalizing and externalizing symptomatology remain insufficiently understood. Furthermore, the majority of prior research has focused primarily on internalizing outcomes (e.g., anxiety, depression) or positive indicators of well-being (e.g., optimism, calmness) (36, 38, 39). Far fewer studies have examined externalizing behaviors (e.g., aggression, impulsivity), despite evidence that males are more likely to manifest mental health challenges through externalizing symptom patterns, potentially leading to under-detection of distress (20, 40). Moreover, limited research has examined whether structural relations among sport participation, competence, and mental health operate similarly across genders.

To address these gaps, the present study examined associations among sport participation, perceived sport competence, perceived social competence, and internalizing and externalizing behaviors in a sample of U.S. adolescents aged 13–18. Specifically, we tested a mediation model in which sport participation was expected to be positively associated with sport and social competence, which, in turn, were hypothesized to be negatively associated with internalizing and externalizing symptomatology.

### Hypothesis

Greater sport participation will be positively associated with sport and social competence, which will, in turn, be negatively associated with internalizing and externalizing symptomatology; these relations are expected to be invariant across female and male adolescents.

## Methods

### Sample

A total of 628 high school students provided the data for the study. This included 311 females and 317 males. Ages ranged from 13 to 18 years with an average age of 14.55 years (*SD* = 1.44). The majority of students self-identified as White/Caucasian (83.10%) and indicated that they did not speak another language at home besides English (78.6%). Further, 60.99% of the students indicated that they participated on an organized sport team.

### Procedures

Data for this study was derived as part of a larger school-wide needs assessment for high schools in the United States using the Community and Youth Collaborative Institute's School Experience Surveys (CAYCI-SES) (29, 41, 42). The CAYCI-SES Compendium of tools has evolved over the years to include surveys for students, parents/caregivers, teachers/school staff, and community stakeholders [see (29), for an accessible overview of the CAYCI-SES's evolution]. For a broader look at the CAYCI mission and links to the survey scale compendium, follow this link: https://cayci.osu.edu/. The CAYCI-SES secondary version includes multiple subscales related to students' experiences at school, competence and well-being, involvement in out-of-school-time activities (including sport), and other factors. For this study, we included items assessing sport participation, the sport and social competence scales, and indicators of mental health. All survey measures were collected using online survey links (e.g., Qualtrics). Youth were included in the final sample if they responded to all the following questionnaire items/measures. The entire assessment took approximately 30 min to complete. All procedures were approved by the Ohio State University's Institutional Review Board.

### Measures

#### Sport participation

Sport participation was measured using three items from the CAYCI-SES. The first item asked youth whether they played on a sport team. Response options included not involved at all, involved to some extent, and actively involved. Youth were also asked how often they participated in sport, with response options scored on a 6-point scale ranging from never to almost every day. Participation was also assessed by asking many days did you have a practice or game for a sport team this week, with scores ranging from 0 to 7 days. Higher scores on each item reflected greater sport participation.

#### Mental health

Two subscales on the CAYCI-SES assess behavioral mental health symptomatology. The Internalizing Behavior scale of the CAYCI-SES (43) measures the extent to which students report inhibited behaviors and feelings that are dealt with internally, rather than by acting them out in the home or school. Participants ranked the degree to which they experienced certain feelings in the past week. Example items include, “In the past week, I felt sad”, “In the past week, I felt lonely”, or “In the past week, I felt worried”. All five items used a five-point Likert response scale, ranging from 1 (*Strongly Disagree*) to 5 (*Strongly Agree*).

The Externalizing Behaviors scale on the CAYCI-SES (42) measures the extent to which youth report behaviors directed outward, such as poor attention or impulsivity, within the current school year. Example items include, “Have you ever gotten in trouble in class?” “Have you been in a fight?”, or “Has your school called home because you were in trouble for your behavior?” All three items used a five-point Likert response scale, ranging from 1 (*Never*) to 5 (*Very Often*).

### Sport and social competence

Two subscales are included on the CAYCI-SES, which are stand-alone tools developed and tested in other sport research to measure perceived competence. The Perceived Social Competence Scale II (43) assessed the degree to which youth engage in prosocial behaviors that allow them to successfully create and maintain positive social interactions with others. Example items include, “I help other people”, “I show concern for others”, “I show care for others”, and “I give support to others”. All four items used a five-point Likert response scale, ranging from 1 (*Not at all true*) to 5 (*Really True*). This tool has been used in other sport research exploring outcomes associated with sport participation (44–46).

Sport competence was measured using the CAYCI-SES and the Self-Perception of Sport Competence Scale (47), which assesses how good, able, and skilled youth perceive themselves to be in sport. Sport competence is measured by three items, including “How good do you think you are at sport”, “How much ability do you think you have?” and “How skilled do you think you are at your sport”. All items used a five-point Likert response scale, ranging from 1 (*Not at all good*) to 5 (*Very skilled*).

### Overview of data analysis

Structural equation analyses using M*plus* 8.1 explored the relationships among variables. Preliminary analyses involved examining the measurement properties of the scales using standard CFA procedures (48). This included testing whether the measurement properties were invariant across males and females. In the main analysis, we tested a series of structural models to determine the effects of sport participation on internalizing and externalizing behaviors, and whether those effects were mediated by perceived sport and social competence.

The data were input as raw scores, and maximum likelihood estimation with robust standard errors (MLM) was used in all model testing. We relied on multiple fit indices to evaluate the adequacy of the estimated models. An acceptable fit of a model was defined by the following: non-significant Satorra-Bentler scaled *χ*2 at *p* < .01; root mean square error of approximation (RMSEA) ≤ .06; comparative fit index (CFI) ≥ .95; and the Standardized Root Mean Square Residual (SRMR) ≤ .08 [see (49)]. We also examined the modification indices to determine whether any local areas of strain were affecting the acceptability of the models.

When evaluating the adequacy of specific invariance constraints explored in the data, we examined changes in CFI and the results of Satorra-Bentler scaled *χ*^2^ difference tests between the progressively constrained and baseline models. A change in CFI ≤ .01 (50) and a non-significant reduction in *χ*^2^ at *p* < .01 served as our guidelines for determining the tenability of the proposed invariance constraints.

## Results

### Preliminary analyses

The univariate skewness and kurtosis values for the 18 observed variables (items) ranged from −1.16 to 2.60 and −1.79 to 6.66, respectively. Given the relatively high kurtosis values on some of the items, we used maximum likelihood estimation with robust standard errors (MLM), as this estimation method is more robust to non-normality (48).

We began by specifying the relationships among the 18 observed and 5 latent variables using CFA to determine whether the measurement structure of the variables was adequate and equivalent across genders. We followed the basic procedures outlined by Brown (48), where a series of increasingly restrictive models were tested and compared. Specifically, we first tested the CFA model for males and females separately. This was followed by testing the groups simultaneously to establish configural invariance (i.e., equal form/factor structure), weak invariance (i.e., equality of factor loadings), and then strong invariance (i.e., equality of item/indicator intercepts). While optional according to Brown (48), we also tested for strict invariance (i.e., equality of item uniqueness). Establishing strong invariance was our desired outcome, as this level of measurement invariance provides reasonable evidence that the students' gender did not influence their responses to the measures in a meaningful way. In all models tested, the 18 observed variables (items) were specified to load on one of the 5 latent constructs (i.e., sport participation, sport competence, social competence, internalizing behaviors, externalizing behaviors) and correlations among the latent variables were freely estimated.

The results of the model testing are shown in Table 1. To begin, both single group solutions provided a good fit to the data based on set of fit indices. While not presented at this point, results indicated that all items significantly and positively loaded on their respective latent constructs in each analysis. As seen in Table 1, there was also reasonable evidence of measurement invariance between genders. First, acceptable fit statistics were examined for all four models tested in this step. More importantly, though, there was a non-significant increase in the Satorra-Bentler scaled *χ^2^*and a change in CFI < .01 moving from the configural invariance model to the weak invariance model and again to the strong invariance model. As noted, this was the level at which we felt the measurement properties were reasonably the same for both males and females. Again, results also showed that the items significantly and positively loaded on their respective latent constructs, and no major local areas of strain were noted.

**Table 1:** Model fit statistics for tests of gender invariance on the set of 18 indicators.

Model Tested	ML *χ*^2^	SB *χ*^2^	*df*	*Δdf*	*SDCS*	RMSEA	SRMR	CFI	Pass?
Single group solutions									
Males (*n* = 317)	193.79**	169.71**	125	–	–	.034	.041	.986	–
Female (*n* = 311)	231.83**	211.69**	125	–	–	.047	.050	.972	–
Measurement invariance									
Configural Invariance	425.62**	380.53**	250	–	–	.041	.045	.979	–
Weak Invariance	447.99**	399.74**	263	13	19.23	.041	.051	.978	Yes
Strong Invariance	475.51**	425.82**	276	13	26.57	.042	.052	.976	Yes
Strict Invariance	597.79**	518.15**	294	31	87.04**	.049	.059	.964	No
Population heterogeneity									
Equal Factor Variance	489.40**	438.21*	281	5	12.38	.042	.059	.975	Yes
Equal Factor Covariances	504.44**	450.96**	291	10	12.89	.042	.065	.974	Yes
Equal Factor Means	608.07**	545.75**	296	5	120.77**	.052	.088	.960	No

MLχ^2^ = Maximum likelihood χ^2^; SB χ^2^ = Satorra-Bentler scaled χ^2^; df, degrees of freedom; SDCS, Satorra-Bentler scaled difference in χ^2^ test statistic; RMSEA, root mean square error of approximation; SRMR, standardized root mean square residual; CFI, comparative fit indices; Pass?, meets criteria to be considered invariant.

* *p* = <.01.

** *p* = <.001.

After establishing strong measurement invariance, Brown (48) suggests exploring aspects of population heterogeneity for descriptive purposes by testing for equal factor variances, equal factor covariances, and equal factor mean scores. The results from these tests are shown in Table 1. In comparison to the strong invariance model, setting the variance of the latent factors to be equal across groups did not result in a meaningful decrease in model fit based on the change in *χ*2 and CFI. The same was found when constraining the factor covariances to be equal across groups. Thus, the variances and covariances of the latent factors were the same for males and females.

There was not, however, support for the invariance of factor means. Follow up explorations, where the factor means for males were fixed to 0 and the means for females were freely estimated, revealed significant (*p* < .05) differences in all the latent variables except for sport participation (females = −.03, *p* = .69). Specifically, female students reported higher social competence (females =  + .19) and internalizing behaviors (females =  + .60) and lower sport competence (females = −.25) and externalizing behaviors (females = −.57) compared to the male students. To review, there was strong evidence for measurement invariance across genders. Given this and particularly the fact that the relationships among the variables were the same for males and females, we decided to include males and females together in the main analyses. This was done to increase the power of the tests and simplify the presentation of the results.

### Main analyses

To determine the effects of sport participation on internalizing and externalizing behaviors, and whether those effects were mediated by perceived sport and social competence, we tested and compared two structural models. In both models, we specified that social competence and sport competence influence both internalizing and externalizing behaviors. Further, the correlations between social and sport competence and between internalizing and externalizing behaviors were freely estimated. The difference between the models involved the estimated relationships between sport participation and the mental health indicators. In the non-mediational model, we specified that sport participation not only indirectly influenced internalizing and externalizing behaviors through the effects on sport and social competence, but also directly influenced these behaviors. In the hypothesized mediational model, however, sport participation had no direct effect on internalizing and externalizing behaviors. Instead, the only effect of sport participation on the mental health indicators was indirect through the mediating variables of sport and social competence.

A summary of the fit associated with these structural models is presented in Table 2. Both models fit the data well (i.e., non-significant *χ*^2^; RMSEA ≤ .08; SRMR ≤ .08, CFI ≥ .95). The comparison of the nested models supported the conclusion that the hypothesized mediational model was a more parsimonious and appropriate representation of the data. Specifically, removing the direct effects of sport participation on internalizing and externalizing behaviors freed 2 *df* and resulted in Satorra-Bentler scaled difference in *χ*^2^ test statistic of 9.05, which was non-significant at *p* < .01.

**Table 2:** Model Fit statistics for testing the hypothesized mediational model.

Model Tested	ML χ^2^	SB χ^2^	*df*	Δ*df*	*SDCS*	RMSEA	SRMR	CFI
Non-Mediational Model	302.46**	269.31**	125	–	–	.043	.042	.977
Hypothesized Mediational Model	311.35**	277.77**	127	2	9.05	.043	.044	.976

MLχ^2^ = Maximum likelihood χ^2^; SB χ^2^ = Satorra-Bentler scaled *χ*^2^; *df*, degrees of freedom; SDCS, Satorra-Bentler scaled difference in χ^2^ test statistic; RMSEA, root mean square error of approximation; SRMR, standardized root mean square residual; CFI, comparative fit indices.

* *p* < 0.01.

** *p* = <.001.

**Figure 1:**
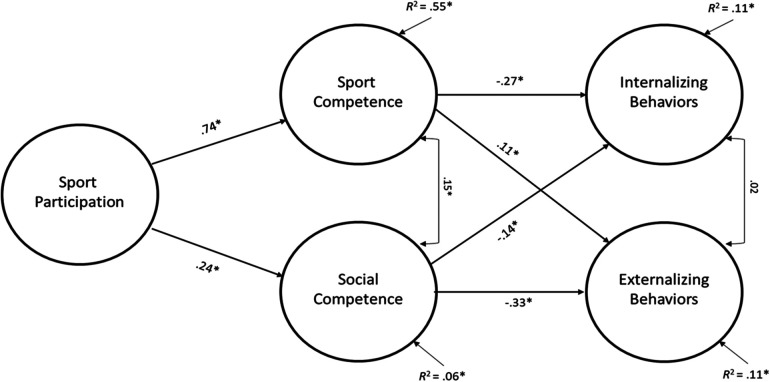
Structural equation model predicting the influence of sport participation on indicators of competence and mental health. All values reflect standardized parameter estimates; *N* = 628; *p = <.01.

The full results of the hypothesized mediational model are presented in Table 2 and Figure 1. First, the measurement portion of the model is included in Table 3. All items significantly (*p* < .01) loaded on the appropriate latent variable, with standardized factor loadings ranging from .64 to .98. Squared multiple correlations ranged from .41 to .88, with an average of .64. The composite reliability estimates ranged from .79 to .96 and average extracted variance estimates ranged from .51 to .92 which both support the internal consistency of the factors. Figure 1 presents the relationships among the key constructs. All parameter estimates are presented in standardized form. The curved arrows reflect correlations between variables, whereas straight lines represent the direct effect of a predictor variable on a criterion variable. All relationships are significant (*p* < .01), apart from the correlation between internalizing and externalizing behaviors. Sport competence and social competence were significantly negatively associated with internalizing and externalizing behaviors. In other words, those reporting higher perceived competence exhibited lower levels of internalizing and externalizing behaviors. Sport participation was a positive and significant predictor of both forms of competence, with the effect on sport competence particularly high (β = .74). The amount of variance explained (*R*^2^) in each of the criterion variables was significant (*p* < .01). The model accounted for the greatest amount of variance in sport competence (*R*^2^ = .55), followed by internalizing (*R*^2^ = .11) and externalizing behaviors (*R*^2^ = .11), and finally social competence (*R*^2^ = .06).

**Table 3:** Standardized parameter estimates from the measurement portion of the hypothesized mediational model.

Variable	Factor loading	Item uniqueness	Squared multiple correlation
Sport participation (ω = .85, AVE = .65)			
1. I play on a sports team.	.84	.30	.70
2. During the past 7 days, on how many days did you have a practice or game for a sport team?	.68	.54	.47
3. How often do you participate in sport, in general?	.89	.20	.80
Sport competence (ω = .96, AVE = .92)			
1. How good do you think you are at sports?	.96	.09	.92
2. How skilled do you think you are at sports?	.98	.04	.96
3. How much ability do you think you have at sports?	.94	.12	.88
Social competence (ω = .86, AVE = .51)			
1. I respect others.	.72	.48	.52
2. I am responsible.	.72	.48	.52
3. I work well with others.	.74	.45	.55
4. I am a good friend.	.67	.55	.45
Internalizing behaviors (ω = .88, AVE = .62)			
1. In the past week, I felt sad.	.92	.15	.85
2. In the past week, I felt lonely.	.86	.26	.75
3. In the past week, people were not nice to me.	.64	.59	.41
4. In the past week, I felt worried.	.81	.34	.65
5. In the past week, I had trouble sleeping.	.66	.56	.44
Externalizing behaviors (ω = .79, AVE = .56)			
1. Have you ever gotten in trouble in class?	.80	.37	.64
2. Has your school called home because you were in trouble for your behavior?	.79	.38	.62
3. Have you ever been in a fight?	.64	.59	.41

ω = composite reliability of the latent variable; values ≥.70 indicate acceptable reliability and ≥.80 indicate good reliability. AVE, average variance extracted; values ≥.50 indicate adequate convergent validity.

Results also revealed that the indirect effect of sport participation on internalizing behaviors was significant (*p* < .01), with a standardized total effects estimate of −.23. This effect was primarily the result of the indirect effect through sport competence, which was significant (−.20, *p* < .01), whereas the indirect effect through social competence was non-significant (−.03, *p* > .01). The effect of sport participation on externalizing behaviors showed a different pattern of results. The total effect of sport participation on externalizing behaviors was non-significant (.03, *p* > .01). This was the case even though the indirect effect through both sport competence (.11) and social competence (−.08) were both significant (*p* < .01).

## Discussion

This study examined the relationships among sport participation, sport and social competence, and mental health outcomes in a sample of U.S. adolescents. Sport participation was positively associated with both sport and social competence, and both forms of competence were negatively related to internalizing behaviors. Sport and social competence also were related to externalizing behaviors, though the overall indirect effect of sport participation on externalizing behaviors was limited. Notably, sport competence showed the strongest association with mental health symptomatology, highlighting its central role in the pathways connecting sport participation to adolescent well-being. Additionally, the indirect pathway from sport participation to internalizing behaviors was primarily mediated by sport competence rather than social competence. Findings suggest that adolescents' perceptions of their athletic abilities are particularly relevant for anxiety and depression outcomes. Sport participation may influence internalizing behaviors through multiple mechanisms, including biological processes (51), cognitive and emotional regulation (24, 52), and interpersonal support from coaches and peers (7), all of which have been associated with anxious and depressive symptoms (53, 54). These findings highlight sport competence as a key factor linking sport participation to mental health.

Unlike the pattern observed for internalizing behaviors, there was limited evidence for both direct and indirect effects of sport participation on externalizing behaviors. Although sport and social competence were negatively related to externalizing behaviors, the overall contribution of sport participation to these outcomes was less pronounced, suggesting that other factors may indeed play a larger role in shaping externalizing behaviors among adolescents.

Examining gender differences in the measurement properties of the models revealed largely similar patterns of relationships between the latent variables for males and females. However, differences in means indicated that females reported higher social competence, lower sport competence, higher internalizing behaviors, and lower externalizing behaviors compared to males. Tentatively, this illustrates the importance of sport participation for female adolescents, particularly in enhancing sport competence, which was the most significant predictor of pathways to reduced internalizing behaviors (of which females were high on). Strengthening sport competence in female participants may therefore be a particularly relevant strategy for supporting positive mental health. Speculatively, strengthening sport competence in female adolescents may be a key pathway to support better mental health. By increasing confidence, mastery, and skill, it can reduce internalizing symptoms, promote resilience, and encourage sustained engagement in physical activity, thus creating long- and short-term benefits.

Collectively, some of these results reinforce findings in neighboring literature. For example, some studies have suggested stronger relationships between sport participation and anxiety in male-dominated samples, as well as benefits for boys' depressive symptoms (12, 55, 56), while other research indicates that organized sport supports girls' anxious and depressive symptoms (55). Taken together, these results suggest that sport participation may promote competence and mental health in all genders, with relevance for addressing internalizing behaviors among female adolescents.

## Implications

The findings of this study have several important implications for youth sport programming, coaching practice, and mental health promotion in school-based sport contexts. Most notably, the results indicate that the relationship between sport participation and adolescent mental health operates primarily through perceived sport and, to a lesser extent, social competence. This suggests that sport participation alone is insufficient to promote positive mental health outcomes. Rather, the quality of the sport experience (i.e., *what* is delivered, *where* it is delivered, *who* delivers it and perhaps most importantly, *how* it is delivered) and its capacity to foster competence appear to be critical mechanisms that have the capacity to facilitate change.

From a practical standpoint, these findings highlight the importance of intentionally designing sport environments (underpinned by research-informed curricula) that prioritize the development of sport competence. Specifically, because sport competence showed the strongest associations with reductions in internalizing symptoms such as anxiety and depression, coaching practices that emphasize mastery, skill acquisition, and clear performance feedback may be particularly beneficial (57). Interestingly, linear, mastery-oriented approaches (e.g., structured practice, repetition, and progressive skill development) may help athletes build confidence in their abilities, thereby supporting psychological well-being (58). Therefore, coaches and sport organizations should be encouraged to consider and (appropriately) implement instructional strategies that focus on improvement and effort rather than solely on outcomes or competition.

Equally, the results demonstrate the value of social competence as a plausible pathway to improved mental health, particularly with respect to externalizing behaviors. Sport settings that emphasize collaboration, communication, and shared problem solving may be especially effective in fostering social skills that translate to better behavioral regulation (59). This finding supports the use of pedagogical approaches that are more experiential and non-linear, allowing athletes to learn through interaction, decision-making, and reflection.

On balance, the above recommendations reflect the complexity of how best to coach (60). While mastery-oriented, structured practices may be especially effective for developing athletic competence and reducing internalizing symptoms like anxiety and depression, experiential, collaborative methods appear valuable for enhancing social competence and addressing externalizing behaviors. Perhaps, rather than viewing one approach as 'superior', it may be more appropriate to consider the specific needs of athletes and the goals of the sport environment (i.e., the contextual features), using a flexible combination of both linear skill development and interactive social learning to promote overall mental health and well-being.

The implications of this research are especially relevant in light of growing evidence that student-athletes experience substantial stress related to sport and academic demands (13, 15) yet are less likely to seek professional mental health support compared to their non-athlete peers. Given the high participation rates in school-based sport, these contexts represent a valuable and (arguably) underutilized setting for preventive mental health initiatives. Put simply, structured, competence-focused sport programs may serve as accessible platforms for fostering resilience, adaptive coping, and help-seeking behaviors among adolescents.

Findings also point to the need for balance in youth sport systems. While developing competence appears to be beneficial, prior research suggests that excessive training demands, high-performance pressures, and elite-level competition may compromise mental health (14). This raises important practical questions regarding the threshold at which sport involvement shifts from being supportive to potentially harmful. Sport stakeholders should be attentive to training volume, performance expectations, and athlete well-being, particularly during critical developmental periods.

## Future directions

Building on our findings, several avenues for future research warrant further consideration to enhance our understanding of sport participation and adolescent mental health. First, studies should explore variations in sport engagement, including frequency, duration, and sport type. There is evidence that sustained participation and team-based contexts may enhance mental health benefits through repeated exposure to positive environments and opportunities for skill development, social interaction, and mastery (30, 61, 62). Expanding measures to capture these multidimensional aspects of sport could clarify how different experiences shape outcomes.

Second, attention should be given to participant characteristics and moderating factors. For example, gender, age, socioeconomic status, and neighborhood resources all appear to influence how sport participation relates to competence and mental health (63). For instance, girls may benefit most in reducing internalizing symptoms through sport competence, while boys may show a reduction in depressive symptoms (12, 55, 56). Similarly, younger adolescents may gain more in collaboration and open-mindedness, whereas older adolescents show stronger improvements in emotional regulation (36).

Third, future research could investigate different levels of sport engagement, including elite vs. recreational participation. Elite athletes often engage in advanced self-regulation and interpersonal skills, which may interact with mental health outcomes (26, 64). Exploring the organization and categorization of sports, school-based vs. recreational programs, and year-round vs. seasonal participation could reveal important mechanisms through which sport contributes to well-being (65).

Longitudinal designs would also provide a greater level of insight into how sport participation, competence, and mental health develop over time. Understanding this temporal element could inform interventions aimed at sustaining engagement, building competence, and targeting mental health outcomes across adolescence. This recommendation also supports the notion that coaches need time with athletes to develop meaningful relationships which are likely to contribute to more positive athlete mental health outcomes (66). These directions present opportunities for sport participation to be engaged as a viable pathway for supporting adolescent mental health, while identifying the specific conditions and populations for which it is most effective.

## Limitations

As in any research, limitations inevitably exist, which may help to shape the direction of future research. First, all measures were based on self-report from adolescents, which may be influenced by recall biases and social desirability. While self-report is commonly used in youth sport research, future studies could incorporate multi-informant data from parents, coaches, or teachers to provide a more comprehensive and objective assessment of sport participation, competence, and mental health outcomes (33).

Second, the study did not specifically examine elite or highly competitive athletes, which limits the generalizability of the findings. Previous research suggests that mental health outcomes may differ for athletes engaged in high-level competition, where stress, training load, and performance pressure can be greater (67). Additionally, the measure of sport participation was assessed via three items and did not capture important contextual factors such as early specialization, frequency, intensity, type of sport (team vs. individual), or competitive level. As early specialization has been linked to increased risk for mental health difficulties, future research should incorporate more sophisticated measures of sport engagement to explore these relationships.

Additionally, the current sample, while adequate for testing the mediational model, was not fully nationally representative. Future studies may wish to include broader and more diverse adolescent populations, as well as examine potential moderators such as age, socioeconomic status, and cultural context, to better understand under what conditions (and for who) sport participation supports mental health. Specifically, it will be important to explore moderators to explore further relationships across different subgroups of adolescents. Qian et al's research supports this contention (36), finding that the strength of the relationships among sport participation and mental health was dependent upon different social-emotional skills (e.g., emotional regulation, collaboration) perceived by gender, age, and socio-economic status of athletes. Continuing to study the relationships with a more diverse sample might afford a better understanding of these relationships.

Finally, although the current findings support a model in which sport participation influences mental health indirectly via competence, longitudinal research is needed to determine the temporal relationships between sport involvement, competence development, and mental health outcomes (12, 30).

## Conclusion

Findings provide additional evidence to support the value of sport participation for addressing mental health symptomatology present among adolescents today. Direct effects were found demonstrating relationships among sport participation and sport and social competence, as well as sport and social competence and internalizing and externalizing behaviors. Additionally, findings point to the relevance of sport competence as a mediator of the relationship between sport participation and internalizing behaviors, in particular, demonstrating further how sport as a developmental context can support adolescent mental health in positive ways.

## Data Availability

The data analyzed in this study is subject to the following licenses/restrictions: Secondary data from schools. Requests to access these datasets should be directed to cayci@osu.edu.
